# Genome-Wide Analysis of Alternative Splicing (AS) Mechanism Provides Insights into Salinity Adaptation in the Livers of Three Euryhaline Teleosts, including *Scophthalmus maximus*, *Cynoglossus semilaevis* and *Oncorhynchus mykiss*

**DOI:** 10.3390/biology11020222

**Published:** 2022-01-30

**Authors:** Yuan Tian, Qinfeng Gao, Shuanglin Dong, Yangen Zhou, Han Yu, Dazhi Liu, Wenzhao Yang

**Affiliations:** 1 Key Laboratory of Mariculture, Ministry of Education, Ocean University of China, Qingdao 266003, China; tianyuan@ouc.edu.cn (Y.T.); dongsl@ouc.edu.cn (S.D.); zhouyg@ouc.edu.cn (Y.Z.); yh9500@stu.ouc.edu.cn (H.Y.); LDZ@stu.ouc.edu.cn (D.L.); yangwenzhao@ouc.edu.cn (W.Y.); 2 Function Laboratory for Marine Fisheries Science and Food Production Processes, Qingdao National Laboratory for Marine Science and Technology, Qingdao 266100, China

**Keywords:** alternative splicing, salinity adaptation, teleosts, RNA-Seq, RNA splicing

## Abstract

**Simple Summary:**

Alternative splicing (AS) is a key post-transcriptional regulatory mechanism that acts an important regulator in response to environmental stimuli in organisms. In the present study, 18 RNA-Seq datasets were utilized to investigate the potential roles of AS in response to different salinity environments in the livers of three euryhaline teleosts, including turbot (*Scophthalmus maximus*), tongue sole (*Cynoglossus semilaevis*) and steelhead trout (*Oncorhynchus mykiss*). The results indicated that different salinity environments changed the splicing patterns of numerous RNA splicing regulators, which might affect the splicing decisions of many downstream target genes in response to salinity changes. This study provides preliminary evidence for the important roles of AS events in salinity adaptation in teleosts.

**Abstract:**

Salinity is an important environmental factor that directly affects the survival of aquatic organisms, including fish. However, the underlying molecular mechanism of salinity adaptation at post-transcriptional regulation levels is still poorly understood in fish. In the present study, 18 RNA-Seq datasets were utilized to investigate the potential roles of alternative splicing (AS) in response to different salinity environments in the livers of three euryhaline teleosts, including turbot (*Scophthalmus maximus*), tongue sole (*Cynoglossus semilaevis*) and steelhead trout (*Oncorhynchus mykiss*). A total of 10,826, 10,741 and 10,112 AS events were identified in the livers of the three species. The characteristics of these AS events were systematically investigated. Furthermore, a total of 940, 590 and 553 differentially alternative splicing (DAS) events were determined and characterized in the livers of turbot, tongue sole and steelhead trout, respectively, between low- and high-salinity environments. Functional enrichment analysis indicated that these DAS genes in the livers of three species were commonly enriched in some GO terms and KEGG pathways associated with RNA processing. The most common DAS genes work as RNA-binding proteins and play crucial roles in the regulation of RNA splicing. The study provides new insights into uncovering the molecular mechanisms of salinity adaptation in teleosts.

## 1. Introduction

Salinity is one of the most important environmental factors, and it greatly affects the survival, growth, development, reproduction, metabolism and other physiological activities of teleosts [1,2]. To cope with salinity changes, many euryhaline teleosts have evolved complex physiological strategies that involve a series of various biological processes [3,4,5]. With the development of high-throughput sequencing technology, RNA-Seq has been regarded as a powerful tool to interpret the functional genomic elements and uncover the molecular mechanisms in organisms [6,7]. 

Over the past few years, RNA-Seq analyses associated with salinity adaptation have been realized in some teleosts, such as striped catfish (*Pangasianodon hypophthalmus*) [7], tongue sole (*Cynoglossus semilaevis*) [8], spotted sea bass (*Lateolabrax maculatus*) [9], steelhead trout (*Oncorhynchus mykiss*) [10] and turbot (*Scophthalmus maximus*) [11]. However, most of these studies were only focused on the differentially expressed transcripts or genes after salinity changes. The knowledge regarding roles of post-transcriptional regulation mechanism in salinity adaptation has been quite limited until now.

Alternative splicing (AS) is a key post-transcriptional regulatory mechanism that produces various splicing variants from same pre-mRNA, making great contributions to increase the transcriptome complexity and enhance proteome diversity in eukaryotes [12,13]. Genome-wide studies have reported that ~95% of multi-exon genes in human (*Homo sapiens*) undergo AS events [14] compared with ~63% of genes in mouse (*Mus musculus*) [15] and ~61% in thale cress (*Arabidopsis thaliana*) [16]. Of them, some genes could even produce hundreds of spliced variants. For instance, there are 247 unique spliced variants derived from the *neurexin-1-alpha* gene in mouse [17]. 

Based on different splicing patterns, AS events can be roughly classified into five basic types, including exon skipping (ES), intron retention (IR), mutually exclusive exon (MXE), alternative 5′ splice site (A5SS) and alternative 3′ splice site (A3SS). In general, ES is the most frequent AS event in animals, while IR is the most prevalent type of AS events in plants [13]. The insertion or deletion of alternative fragments in mRNAs is likely to directly change the protein products and heavily affect the biological characteristics, such as the structure, function, stability and subcellular localization [13,18]. 

The two AS isoforms from the *Bcl-x* gene in fruit flies (*Drosophila melanogaster*) have been proven to participate in activating and inhibiting apoptosis [19]. AS could also modulate the abundance of mRNA via introduction of premature termination codons (PTCs). The mRNA with PTCs would be recognized by specific proteins and degraded by nonsense-mediated decay pathways in cells [20,21,22].

Mounting evidence has revealed that AS acts an important regulator in response to environmental stresses in teleosts. It was reported that the exon 9a of *heat shock transcription factor 1* gene is differentially alternative spliced between control and heat-intolerant groups of channel catfish (*Ictalurus punctatus*) under heat stress, suggesting its potential roles in heat tolerance [23]. In rainbow trout (*Oncorhynchus mykiss*), differentially alternative splicing (DAS) events are significantly increased following heat exposure [24]. Hundreds of DAS events in the heart and brain of Nile tilapia (*Oreochromis niloticus*), enriched in the circadian clock pathway, are proven to be correlated with cold adaptation [25]. Similarly, differences in splicing patterns are observed for genes involved in striated muscle contraction and the mus cle myosin complex, which are thought to be key in the cold adaptation in skeletal muscle of zebrafish (*Danio rerio*), Atlantic killifish (*Fundulus heteroclitus*) and threespine stickleback (*Gasterosteus aculeatus*) (Healy & Schulte, 2019). Under acute hypoxia environment, *elongation factor 2 kinase*, *NADH dehydrogenase (ubiquinone) Fe-S protein 1*, *family with sequence similarity 162 member A* and *NDRG family member 2* genes undergo differential usage of exons, which have been demonstrated to be tightly related to hypoxia tolerance in heart and gill of Nile tilapia [26,27]. 

Numerous AS events are greatly induced (over a 20% increase) by high-salinity environment in both the liver and gills of spotted sea bass [28]. This finding suggests that AS may be an important regulator in response to salinity changes in spotted sea bass. Nevertheless, the common mechanism of AS in salinity adaptation across teleosts has remained unknown. Turbot and tongue sole are typical euryhaline teleosts with the remarkable ability to survive in a wide range of salinity environments from 0 to 50 ppt [8,11,29,30,31,32]. Steelhead trout is also capable of adapting and of living in 0-32 ppt salinity environments [10,33]. Therefore, they provide excellent models to investigate molecular mechanism of salinity adaptation in teleosts. In the present study, AS analysis based on RNA-Seq datasets were performed to investigate the potential roles of various splicing patterns in salinity adaptation in the livers of turbot, tongue sole and steelhead trout. The study not only provides a new insight into uncovering the molecular mechanism of salinity adaptation in teleosts, but also serves as a valuable reference for related studies in the future.

## 2. Materials and Methods

### 2.1. Data Acquisition and Experimental Design

A total of 18 RNA-Seq datasets of livers in turbot, tongue sole and steelhead trout were derived from previously published studies, the objects of which were to investigate quantitative changes in gene expression in response to different salinity environments [8,10,11]. The salinity experiments of each study were briefly described as followed: healthy turbot were obtained and placed in six identical tanks (4000 L) full of seawater (salinity: 30 ± 0.2 ppt) for one-week acclimatization. Then, individuals from three tanks were directly exposed to dechlorinated tap water and regarded as the low-salinity group. The others were maintained in seawater and labeled as the high-salinity group. For both low- and high-salinity group, six individuals per tank were randomly collected and quickly anesthetized with MS-222 at 24 h after salinity changes. The livers were obtained and frozen in liquid nitrogen for following RNA extraction and transcriptome sequencing. For tongue sole, female individuals with similar sizes were selected and acclimatized in six experimental tanks at an optimal salinity of 30 ppt for one week. After that, six tanks were equally divided into low- and high-salinity groups. The salinity of high-salinity group were kept at 30 ppt, while the salinity of low-salinity group was gradually decreased from 30 to 15 ppt at a constant rate of 5 ppt per day for 3 days. After 60-day culture, three individuals per tank were euthanized and anesthetized with MS-222. The livers were immediately removed and frozen in liquid nitrogen for RNA extraction and transcriptome sequencing. For steelhead trout, healthy individuals were collected and reared in six cylindrical tanks under freshwater for two weeks prior to the salinity experiment. After that, low- and high-salinity groups were established in three replicated tanks. The low-salinity group was maintained in freshwater. Simultaneously, the salinity of high-salinity group was directly increased to 14 ppt, followed by a daily increase of 2 ppt until 30 ppt. The whole experiment lasted for 40 days. At the end, three individuals per tank were anesthetized with MS-222. Following anesthesia, livers were quickly dissected and immersed into liquid nitrogen for high-throughput sequencing.

### 2.2. Identification of AS and Differentially Alternative Splicing (DAS) Events

To construct the AS landscapes in the livers of turbot, tongue sole and steelhead trout, a total of 18 RNA-Seq datasets (six datasets from each fish) were selected and analyzed in the present study. The raw data of RNA-Seq derived from NCBI SRA database were converted to FASTQ format using SRA Toolkit v 2.9.0. Quality trimming and adapter clipping were performed to provide clean reads using fastp v0.23.2 with default parameters. The qualities of clean reads was further assessed using FastQC v0.11.9. The reference genomes and their corresponding annotation files of turbot, tongue sole and steelhead trout were also obtained from NCBI database with the assembly number of GCA_013347765.1, GCA_000523025.1 and GCA_002163495.1, respectively. High-quality clean reads of turbot, tongue sole and steelhead trout were then aligned to their corresponding reference genomes using Hisat2 v2.2.1 with default parameters. The aligned results were strictly filtered, and only unique alignments were retained for subsequent analysis. Reference-based transcript assembly was performed using StringTie v2.1.7. AStalavista v4.0 was utilized to automatically characterize the complete landscapes of AS events in the livers of the three species. The five typical AS events were defined by specific codes, including “0, 1-2^” for ES, “0, 1^2-” for IR, “1-2^, 3-4^” for MXE, “1^, 2^” for A5SS and “1-, 2-” for A3SS. 

In addition, rMATs v.4.0.1 was also used to detect the AS events in each fish. Only AS events detected by both AStalavista and rMATs were considered stable for following analysis. Moreover, salt-responsive AS events were also determined using rMATs software by computing the inclusion level from two-group RNA-Seq data with replicates and constructing a hierarchical framework to model the variability among replicates. The threshold of DAS events was set as FDR-adjusted *p*-value < 0.05.

### 2.3. Identification of AS and Differentially Expressed Genes (DEGs) Events

Read matrix were constructed based on filtered alignments using featureCounts v 2.0.1. Then, read counts were transformed into fragments per kilobase of transcript per million fragments mapped (FPKM) for the normalization of gene expression levels. DESeq2 v1.34.0 R package was employed to determine the differentially expressed genes (DEGs) in the livers of all three species under different salinity environments. The significant thresholds of DEGs were set as |log_2_ (fold change)| ≥ 1 and *p*-value < 0.05.

### 2.4. Functional Annotation and Enrichment Analysis

The functional annotations of protein-coding genes in each fish were conducted using eggNOG-mapper v5.0 with diamond search mode. The functional categories of Gene Ontology (GO) terms, and Kyoto Encyclopedia of Genes and Genomes (KEGG) pathways were extracted and used to construct specific Org.db databases using AnnotationForge R package v3.14. Based on these Org.db databases, GO and KEGG enrichment analyses of turbot, tongue sole and steelhead trout were implemented using clusterProfiler v4.2.0 R package, respectively. Additionally, enrichment results were further visualized using the ggplot2 R package.

### 2.5. Orthologues Gene Identification

To obtain the putative orthologs among turbot, tongue sole and steelhead trout, a reciprocal best hit (RHB) strategy was performed using the BLAST v2.12.0 program. Based on the information of annotated gene model, only the longest transcripts of each gene were extracted and translated into amino acid sequences. Then, the predicated amino acid sequences of turbot were used as queries for the BLAST program with the parameters of *e*-value < 1 × 10^−5^. The RHB were performed for each pair of genes between tongue sole and turbot, steelhead trout and turbot. The putative orthologs were identified based on the top BLAST hits in each reciprocal BLAST pair.

## 3. Results

### 3.1. AS Landscapes in Livers of Turbot, Tongue Sole and Steelhead Trout

**Figure 1:**
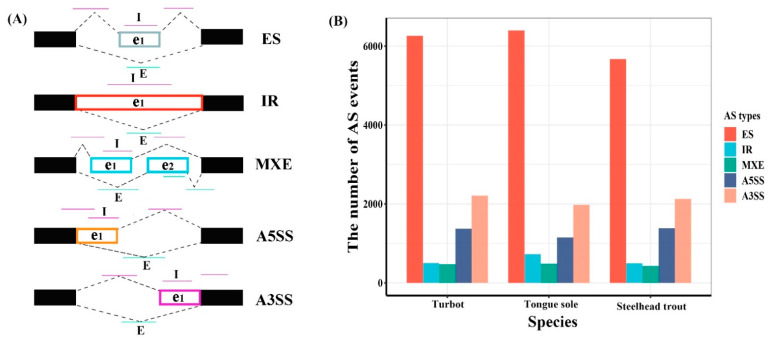
Landscapes of AS events. (**A**) Schematic diagram of five typical AS events. The black solid boxes are used to represent the constitutive exons, while e1 and e2 indicate the alternative spliced fragments. The “I” and “E” letters represent the inclusion and exclusion isoforms, and their reads are marked by carmine and azure lines, respectively. (**B**) The statistics of alternative splicing events in the livers of turbot, tongue sole and steelhead trout.

In total, 10,826, 10,741 and 10,112 AS events were identified in the livers of turbot, tongue sole and steelhead trout, respectively (Supplementary Table S1). These AS events were classified into five basic types, including ES, IR, MXE, A5SS and A3SS (Figure 1A). Among them, ES was the most abundant type, accounting for more than half of AS events in the livers of all three species, followed by A3SS, A5SS, IR and MXE (Figure 1B).

**Figure 2:**
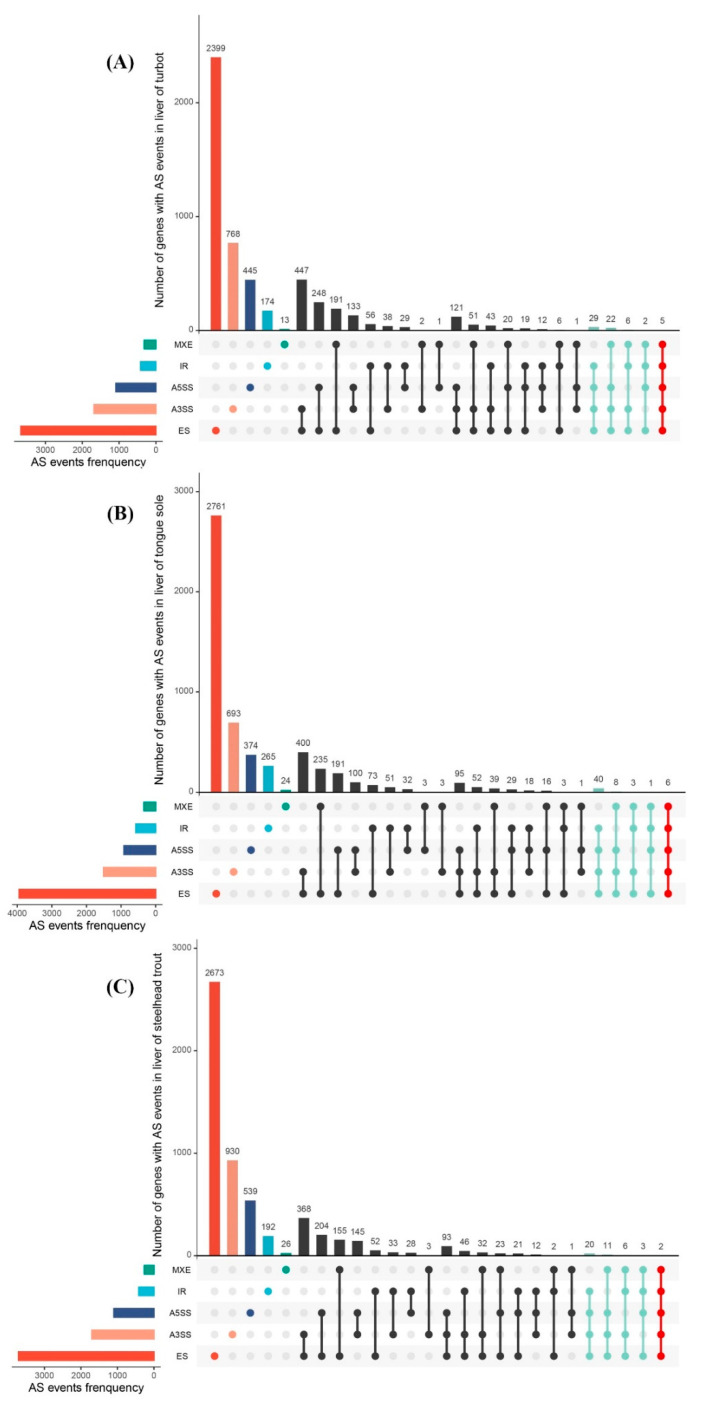
UpSet plot showing the intersections between genes and their AS events in the livers of turbot (**A**), tongue sole (**B**) and steelhead trout (**C**). The upper and left bars were used to represent the number of genes and their AS events. Genes with four and five different typical AS events were marked with azure and red colors.

Additionally, we found that the 10,826, 10,741 and 10,112 AS events were derived from 5281, 5516 and 5614 functional genes, demonstrating that numerous genes may undergo at least two different AS events. The intersections between genes and their AS events in three species were intuitively visualized through the construction of UpSet plots, as shown in Figure 2A–C. The results clearly showed that most genes in turbot, tongue sole and steelhead trout only produced single AS events. Nevertheless, 64 genes in turbot, 58 genes in tongue sole and 42 genes in steelhead trout had the ability to produce greater than three different AS events. 

These functional genes were closely associated with growth, signal transduction, the cell cycle and immune response. Strikingly, five, six and two genes even harbored all five different AS events in three species, including *vegfa*, *pfkfb2b*, *nlrc3*, *p4ha2* and *uncharacterized LOC118318343* in turbot; *sox11*, *hcf1*, *rbms3*, *spo11*, *uncharacterized LOC103399889* and *uncharacterized LOC112487504* in tongue sole; and *sec31* and *pps1* in steelhead trout. Their detailed information is summarized in Supplementary Table S2.

### 3.2. Distribution of AS Events across the Genomes of Turbot, Tongue Sole and Steelhead Trout

**Figure 3:**
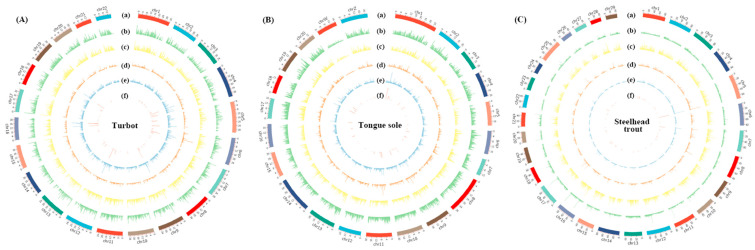
The distribution of protein-coding genes and AS events across the whole genomes of turbot (**A**), tongue sole (**B**) and steelhead trout (**C**). The bar plots from the outer to inner were used to represent the: (**a**) genome karyotype; (**b**) distribution of all the protein-coding genes; (**c**) distribution of protein-coding genes with AS events; (**d**) distribution of all the AS events; (**e**) density of AS events (AS event number/gene number); and (**f**) AS hotspot distribution. The distribution of both genes and AS events were calculated within non-overlap 200 kb bins.

We investigated the distribution of AS events across the whole genomes of turbot, tongue sole and steelhead trout, which were further visualized using Circos plots (Figure 3A–C). To well characterize the distribution of AS events, the density of AS events (AS event number/gene number) was calculated within non-overlap 200 kb bins on the genomes of three species. The average densities of AS events in turbot, tongue sole and steelhead trout were estimated as 2.08, 1.94 and 1.79, respectively.

We also found that the AS event densities varied dramatically across the same chromosome, organized into numerous hotspots with the density of AS events greater than 5. Of the identified hotspots, each gene locating in 15.40–15.60 Mb on Chr12 and 18.60–18.80 Mb on Chr16 of turbot could generate 17 AS events on average. Similar observations were also detected in 13.60–13.80 Mb on Chr18 of tongue sole and 55.40–54.60 Mb on Chr25 of steelhead trout, the AS event densities of which were 12.66 and 11.67, respectively. 

There were 109, 103 and 107 functional genes located in the AS hotspots of turbot, tongue sole and steelhead trout. KEGG enrichment analysis was carried out to explore the potential functions of these genes. The results indicated that genes in turbot were significantly enriched in apoptosis, folate biosynthesis and the complement and coagulation cascades pathways. The genes in tongue sole were closely associated with complement and coagulation cascades, and some disease-related pathways, such as staphylococcus aureus infection and legionellosis. 

Genes in steelhead trout were tightly related to the complement and coagulation cascades, alpha-Linolenic acid metabolism and biosynthesis of unsaturated fatty acids. It is noted that genes located in AS hotspots of all three species were commonly enriched in complement and coagulation cascades, particularly complement cascades. The related elements in complement and coagulation cascades and functional gene information located in AS hotspots are visualized and given in Supplementary Figure S1 and Supplementary Table S3, respectively.

### 3.3. Identification of DAS Events under Different Salinity Environments

DAS events were determined by comparing the RNA-Seq datasets between the low- and high-salinity groups in the livers of all three species. As results, a total of 940, 590 and 553 DAS events were identified in turbot, tongue sole and steelhead trout, corresponding to 769, 482 and 467 functional genes, respectively (Table 1). Among them, 37, 56 and 23 genes showed differentially expression levels and differentially alternative splices in the livers of turbot, tongue sole and steelhead trout (Supplementary Figure S2; Supplementary Table S4). 

**Figure 4:**
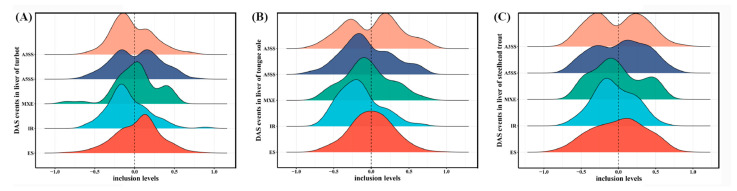
Inclusion level differences of DAS events in the livers of turbot (**A**), tongue sole (**B**) and steelhead trout (**C**) between low- and high-salinity groups. These differences indicate the inclusion levels of low-salinity relative to high-salinity groups.

In agreement with the classification patterns of all AS events, most DAS events in all three species belonged to the ES type, followed by A3SS, A5SS, IR and MXE (Table 1; Supplementary Figure S3). In addition, a pair of shorter and longer isoforms in AS events were defined as inclusion and exclusion by rMATS, respectively (Figure 1A). In the present study, exclusion and inclusion isoforms in each type of DAS events were systematically characterized according to the values of the relative inclusion levels (Figure 4). The results clearly showed that different salinity environments resulted in diverse patterns of exclusion and inclusion isoforms in most AS types, except for IR. 

Global increased inclusion levels and decreased proportions of exclusion isoforms in IR were detected in the livers of all three species under the high-salinity environment (Figure 4). It was indicated that the high-salinity environment could elevate the intron retention levels and promote the selection of longer introns in some differentially spliced genes. Splicing models of several genes were constructed as shown in Supplementary Figure S4. The functional analysis showed that these genes with different IRs were tightly related to the regulation of mRNA 3′-end processing, cap mRNA methylation and protein dilapidation (Supplementary Figure S3).

In order to detect the common DAS genes across species under different salinity environments, the putative orthologs among turbot, tongue sole and steelhead trout were obtained using the RHB strategy. As result, a total of 10 common DAS genes were detected in all three species (Supplementary Figures S5–S7). The functional annotation revealed that these genes were involved in transcription regulation, RNA splicing, cytoskeleton regulation, signal transduction, neuronal development, protein transport, endoplasmic reticulum formation and hyaluronan metabolism (Supplementary Table S5).

### 3.4. Enrichment Analysis of DAS Genes under Different Salinity Environments

**Figure 5:**
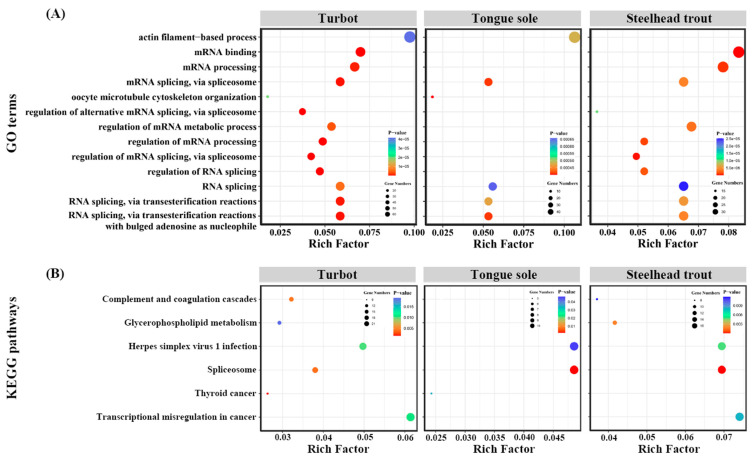
GO (**A**) and KEGG (**B**) enrichment analysis of identified DAS genes in the livers of turbot, tongue sole and steelhead trout under different salinity environments.

Enrichment analysis was performed to explore the potential functions of all the DAS genes in the livers of all three species. These GO terms and KEGG pathways, commonly enriched in at least two fish and ranked in top 30, were displayed in Figure 5. The results clearly indicated that many DAS genes were primarily enriched in some GO terms associated with the biological processes of RNA processing, such as mRNA binding, mRNA processing, mRNA splicing, via spliceosome, RNA splicing, regulation of mRNA metabolic, regulation of RNA splicing and so on (Figure 5A). 

The KEGG enrichment analysis showed that complement and coagulation cascades, glycerophospholipid metabolism and herpes simplex virus 1 infection were also enriched in the livers of turbot, tongue sole and steelhead trout. Moreover, we found that these DAS genes were tightly related to the spliceosome pathway and were in parallel with the results of the GO enrichment analysis (Figure 5B). The detailed information of these enriched GO terms and KEGG pathways are given in Supplementary Tables S6 and S7.

### 3.5. Function Categories of Common DAS Genes Associated with RNA Processing

**Figure 6:**
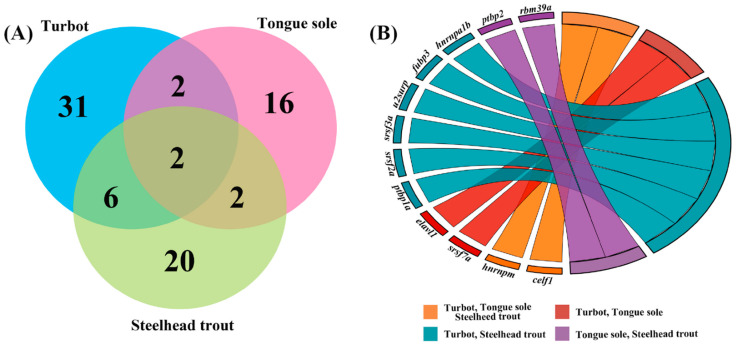
Common DAS genes associated with RNA processing in the livers of turbot, tongue sole and steelhead trout under different salinity environments. (**A**) Venn diagram showing the numbers of common DAS genes among three species. (**B**) GOChord plot of the relationship between the list of common DAS genes and their corresponding species classification. The full names of common DAS genes are given in Supplementary Table S8.

The GO and KEGG enrichment analyses provided considerable evidences that DAS genes associated with RNA processing were crucial in response to different salinity environments. Based on the orthologous relationships, these DAS genes were associated with RNA processing and existed in at least two fish. Their specific biology functions were further investigated by reviewing published documents. As shown in Figure 6, most DAS genes associated with RNA processing appear to be species-specific, while a total of common 12 DAS genes were detected in at least two fish, suggesting important roles in salinity regulation in teleosts. 

**Figure 7:**
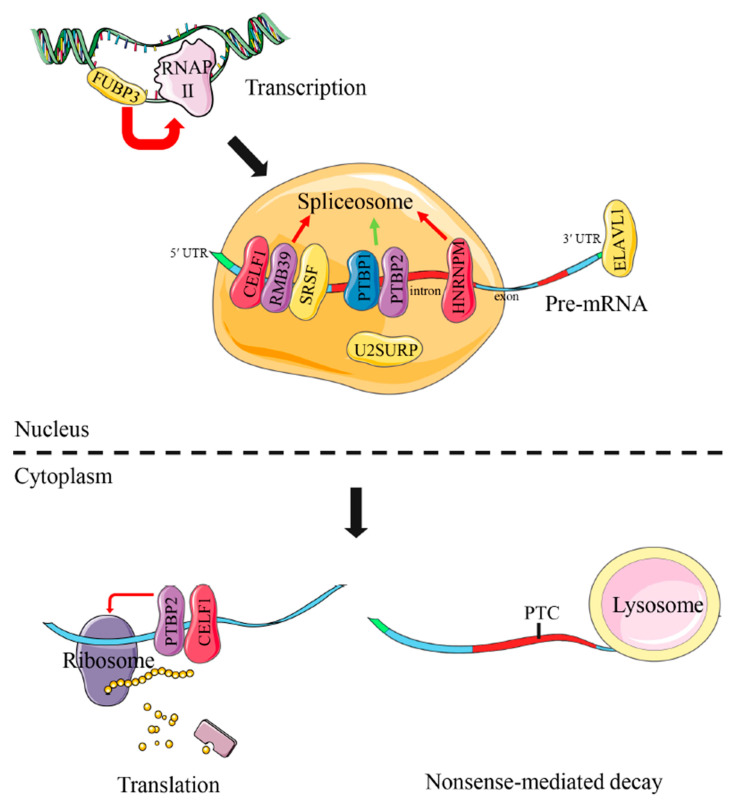
Schematic diagram of common DAS genes associated with RNA processing in the livers of turbot, tongue sole and steelhead trout under different salinity environments. Red and green arrows are used to represent positive and negative regulation function, respectively. The proteins, coded by common DAS genes, are marked by colorful modules. The full names of common DAS genes are given in Supplementary Table S8.

A putative model of functional interaction of these DAS genes was proposed as in Figure 7. Of them, most DAS genes were involved in RNA splicing, including *celf1*, *rmb39*, *ptbp1*, *ptbp2*, *hnrnpm*, *u2surp*, *srsf2a*, *srsf3a* and *srsf7a*. Additionally, *ptbp2* and *celf1* were also implicated in translation regulation. Research reported that *fubp3* could work for transcriptional regulation and *elavl1* for mRNA stability. The specific biology functions of these common DAS genes would be discussed in the next section.

## 4. Discussion

Salinity adaptation is a complicated process that involves multiple physiological responses. In the present study, we conducted a comprehensive study to identify and characterize the AS events associated with salinity adaptation in the livers of turbot, tongue sole and steelhead trout, providing valuable information to better understand the underlying molecular mechanisms of salinity adaptation in teleosts. In fish, the liver is generally considered as an essential metabolic organ, supplying energy to other osmoregulatory organs [1,34]. Furthermore, it has been reported that the liver is also an important antioxidant defense system in response to environmental stresses [35]. 

The present study explored genome-wide AS events in the livers of three species based on the 18 RNA-Seq datasets. Thousands of AS events were identified and characterized, suggesting that AS could be a widespread phenomenon in the livers of the three species. Among these identified AS events, ES was the most frequent type, which is largely consistent with previous observation in teleosts, such as channel catfish [23], rainbow trout [24] and spotted sea bass [28]. As an important post-transcriptional regulation mechanism, AS events could generate different transcripts from the same pre-mRNA. Genes experiencing different AS events can create a much greater extent of transcript diversity [36]. These transcripts may be degraded by nonsense-mediated decay pathways to regulate the transcript abundance or encoding proteins with subtle functional differences that can have profound biological consequences [37]. In the present study, we found that several genes could produce all five AS events in the livers of all three fish, such as *pfkfb2b* in turbot, *sox11* in tongue sole and *sec31* in steelhead trout. As is known, *pfkfb2b* could work not only as a key regulator of glycolysis through catalyzing the synthesis and degradation of fructose-2, 6-bisphosphatase but also could affect the biological process of liver regeneration [38,39]. *Sox11* generally acts as a transcriptional activator to enhance the transcriptional activities of some functional genes [40]. A recent study demonstrated that sox transcription factors are also implicated in liver development [41]. In addition, *sec31* could function in vesicle budding and cargo export from the endoplasmic reticulum, participating in protein transport [42]. The diverse mRNA of these genes may coordinate physiologically meaningful changes in protein isoforms to maintain the normal biological functions of the liver in these fish, such as liver regeneration, development and protein metabolism. However, their specific molecular mechanisms required further study in the future. Additionally, functional genes with diverse AS events were also detected in other organisms, such as the *Na^+^/K^+^/2Cl^−^ cotransporter* in humans [43], *signal transducer and activator of transcription 3* in zebrafish [44], *immunoglobulin heavy chain* in spotted sea bass [9] and *acetyl-CoA carboxylase alpha* in swamp eels (*Monopterus albus*) [45].

Additionally, many hotspots of AS events were also detected across the genomes of turbot, tongue sole and steelhead trout. In all three species, the genes located in AS hotspot regions were commonly enriched in complement and coagulation cascades pathways, particularly the complement cascade system. The complement and coagulation systems are two distinct multi-component protein networks, both of which work as important innate defenses against external threats [46,47,48]. The activated complement system could elicit a pro-inflammatory response and recruit immune cells from both innate and adaptive branches of the immune system, playing crucial roles in the defense against pathogens [49,50,51]. Whereas coagulation is able to culminate the formation of thrombin and convert soluble fibrinogen to insoluble fibrin clots, acting as a main column in hemostasis in organisms [47,52]. Our results have revealed that many key elements, such as C2, C3, C5, C8, C1INH, FH, PLG and CR1, were located in the AS hotspot regions, suggesting that AS events frequently appear in these genes and produce a large number of alternative spliced transcripts. Furthermore, we found that the most abundant type of AS events in these elements is ES. Through changing the array of exons, AS events may affect the encoding sequences of transcripts and increase their proteomic diversity to modulate the functionality of complement and coagulation cascades.

Mounting evidence indicates that the AS mechanism is closely implicated in the response to environmental stimuli in teleosts, such as the heat tolerance of rainbow trout [24] and hypoxia adaptation of Nile tilapia [26]. In the present study, there were substantial changes in the splicing patterns of functional genes in the livers of all three species between low- and high-salinity environments, resulting in numerous DAS events. Of these DAS events, ES is the most common type as expected. IR is greatly induced by high-salinity environment in the livers of all three species. It is known that ES is the most-prevalent type of alternative splicing in animals, which could work as a major contributor to increase the complexity of proteomes [53,54]. Whereas IR is generally considered an aberrant splicing event with little functional consequence [55]. Through coupling to nonsense-mediated decay, IR can be used to govern gene expression levels [56]. Based on the functional analysis, ES and IR events may have affected these genes associated with mRNA binding, cytoskeleton organization, site selection of RNA transcription, mRNA methylation and protein dilapidation in the livers of three species under different salinity environments. Although the functional differences among alternative spliced transcripts remained largely unexplored, the models of the *fubp1*, *srsf2a*, *mark3*, *cmtr1*, *fus*, *atg4b*, *ewsr1b* and *tom1l2* genes clearly showed ES and IR caused the frame shift mutations or inserted premature termination codes. This would result in different protein products or nonsense-mediated decay to influence gene functions in response to different salinity environments in the livers of three species.

The transcribed pre-mRNAs have to undergo intron removal and exon joints to form mature mRNA. Pre-mRNA splicing takes place in the nucleus carried out by spliceosome, a large molecular complex [13]. The spliceosome core is composed of five small nuclear ribonucleoproteins and many splice-related functional proteins, which assemble at introns in a precise order [57,58]. In addition to the core components of the spliceosome, some RNA-binding proteins also play important roles in the determination of splice-site selection in pre-mRNA, such as serine/arginine-rich (SR) splicing factors and heterogeneous nuclear ribonucleoproteins (hnRNPs) [59,60]. In the present study, we found that the many DAS genes in the livers of all three species under different salinity environments were tightly related to RNA processing, especially RNA splicing. This result is largely consistent with previous observations in the salinity adaptation of spotted sea bass [28], heat tolerance of rainbow trout [24] and bacteria defense of channel catfish [61]. There were two common RNA splicing regulators (*celf1* and *hnrnpm*) with DAS events in the livers of all three species under different salinity environments. The functional protein, encoded by *celf1*, is implicated in mediating the inclusion and exclusion of some specific exons in functional genes [62,63]. In addition, *hnrnpm* is reported to be involved in the correct splicing of a subset of pre-mRNA [64]. The other common DAS genes identified in at least two species include *ptbp1*, *ptbp2*, *rmb39*, *u2surp*, *srsf2a*, *srsf3a*, *srsf7a*, *fubp3* and *elval1*, which also act as important splicing factors and take part in the regulation of RNA splicing [63,64,65,66,67,68,69,70,71]. Different salinity environments changed the splicing patterns and resulted in different splicing transcripts of these RNA splicing regulators, which are likely to alter their functions in pre-mRNA splicing and affect the splicing decisions of downstream target genes in response to salinity changes. Although not enriched in GO terms or KEGG enrichments, many DAS genes associated with salinity adaptation were accompanied with differentially expressed levels under different salinity environments, such as *sodium- and chloride-dependent taurine transporter* and *solute carrier family 12 member 7* for ion and amino transport, *fatty acid desaturase 2* and *acyl-coenzyme A thioesterase 1* for the energy metabolism. This indicates that RNA splicing regulators may regulate these functional genes through affecting their transcript abundance. The involvement of these genes indicates their importance in response to salinity changes.

## 5. Conclusions

In the present study, a total of 18 RNA-Seq datasets were used to investigate the potential roles of AS events in salinity adaptation in the livers of turbot, tongue sole and steelhead trout. A total of 10,826, 10,741 and 10,112 AS events were identified in the livers of turbot, tongue sole and steelhead trout, respectively. The characteristics of these AS events were systematically investigated. A total of 940, 590 and 553 DAS events were identified between low- and high-salinity environments in the livers of three species. The results of GO and KEGG enrichment analysis indicated that these DAS genes were tightly related to RNA processing, particularly RNA splicing. The common DAS genes in three species worked as RNA-binding proteins, thereby, playing crucial roles in the regulation of RNA splicing. This finding suggests that different salinity environments significantly changed the splicing patterns of these RNA processing regulators, which, in turn, may affect the AS of downstream target genes in response to salinity changes in teleosts. Taken together, this study provides preliminary evidence for the important roles of AS events in salinity adaptation in teleosts. Further functional studies are needed to elucidate the underlying mechanisms.

## Figures and Tables

**Table 1:** DAS events in the livers of turbot, tongue sole and steelhead trout under different salinity environments. The DAS events were determined using calculating the inclusion levels of low-salinity groups in relative to high-salinity groups in three species. The gene number of each AS type are listed in the brackets.

AS Events	Turbot	Tongue Sole	Steelhead Trout
ES	544 (469)	404 (344)	222 (201)
IR	80 (75)	45 (45)	84 (75)
MXE	47 (38)	32 (26)	40 (33)
A5SS	115 (108)	39 (38)	84 (81)
A3SS	154 (141)	70 (65)	123 (118)
Total	940 (769)	590 (482)	553 (467)

## Data Availability

RNA-Seq data of turbot, tongue sole and steelhead trout can be derived from NCBI SRA database with BioProject accession number of PRJNA656408, PRJNA315357 and PRJNA797919, respectively.

## Supplementary Materials

The following supporting information can be downloaded at: https://www.mdpi.com/article/10.3390/biology11020222/s1, Table S1: Alternative splicing events in livers of turbot, tongue sole and steelhead trout; Table S2: Summary information of functional genes with five typical AS events in livers of all three fishes; Table S3: Information of AS genes associated with complement and coagulation cascades pathways in livers of turbot, tongue sole and steelhead trout under different salinity environments. Species; Table S4: Information of genes with differentially expressed levels and differentially alternative splices in liver of turbot, tongue sole and steelhead trout under different salinity environments; Table S5: Summary of 10 common DAS genes identified in livers of turbot, tongue sole and steelhead trout under different salinity environments; Table S6: GO enrichment analysis of DAS genes in livers of turbot, tongue sole and steelhead trout under different salinity environments; Table S7: KEGG enrichment analysis of DAS genes in livers of turbot, tongue sole and steelhead trout under different salinity environments; Table S8: Detailed information of 12 common DAS genes associated with RNA processing in livers of turbot, tongue sole and steelhead trout under different salinity environments; Figure S1: Complement and coagulation cascades pathway of AS genes located in the hotspot regions in livers of turbot, tongue sole and steelhead trout. AS genes in turbot, tongue sole and steelhead trout were showed with green, purple and yellow boxes, respectively. The common AS genes in both steelhead trout and tongue sole were marked with red boxes. Blue box represented the common AS genes in turbot and steelhead trout; Figure S2: Veen diagrams showing the numbers of common DEGs and DAS genes in livers of turbot (A), tongue sole (B) and steelhead trout (C) under different salinity environments; Figure S3: GO enrichment analysis of different ES (A) and IR (B) genes in livers of turbot, tongue sole and steelhead trout under different salinity environments; Figure S4: Splicing models of differential ES and IR events in livers of turbot (A,B), tongue sole (C,D) and steelhead trout (E,F) under different salinity environments. Filled boxed represented exons and introns were showed using black lines; Figure S5: Venn diagrams showing the numbers of common DAS genes in livers of turbot, tongue sole and steelhead trout under different salinity environments. Figure S6: Splicing models of common DAS genes in livers of turbot, tongue sole and steelhead trout under different salinity environments. Filled boxed represented exons and introns were showed using black lines; Figure S7: Splicing models of common DAS genes associate with RNA splicing in livers of turbot, tongue sole and steelhead trout under different salinity environments. Filled boxed represented exons and introns were showed using black lines.
