# Supplementary material for: Genome-Wide Analysis of Alternative Splicing (AS) Mechanism Provides Insights into Salinity Adaptation in the Livers of Three Euryhaline Teleosts, including Scophthalmus maximus, Cynoglossus semilaevis and Oncorhynchus mykiss
Source: Biology (Basel). 2022 Jan 30;11(2):222. doi: 10.3390/biology11020222 (PMC8869236; doi:10.3390/biology11020222)
Supplement: Supplementary file 1 [file biology-11-00222-s001.zip › biology-1535578 Supplementary materials.pdf]

Supplementary Table S1 Alternative splicing events in livers of turbot, tongue sole and steelhead trout.

Species	ES	IR	MXE	A3SS	A5SS	Total
Turbot	6264	505	476	2209	1372	10826
Tongue sole	6395	729	488	1977	1152	10741
Rainbow trout	5674	499	430	2127	1382	10112
Total	18333	1733	1394	6313	3906	31679

Supplementary Table S2 Summary information of functional genes with five typical AS events in livers of all three fishes

Species	Gene id	location	Nr annotation
Turbot	LOC118284804	Chr16: 18783559-18813251	*vascular endothelial growth factor A* (*vegfa*)
	LOC118318343	Chr12: 10786101-10892744	*uncharacterized LOC118318343*
	pfkfb2b	Chr6: 5464183-5474102	*6-phosphofructo-2-kinase/fructose-2,6-biphosphatase 2b* (*pfkfb2b*)
	LOC118288674	Chr19: 1430104-1441822	*NLR family CARD domain-containing protein 3-like* (*nlrc3*)
	p4ha2	Chr4: 8880859-8903118	*procollagen-proline 2-oxoglutarate 4-dioxygenase* (*p4ha2*)
Tongue sole	LOC103377951	Chr1: 8366172-8802725	*transcription factor SOX-11* (*sox11*)
	LOC103384375	Chr10: 151735-173505	*host cell factor 1* (*hcf1*)
	LOC112487504	Chr9: 7078092-7083544	*uncharacterized LOC112487504*
	LOC103399889	Chr2: 14063571-14073796	*uncharacterized LOC103399889*
	rbms3	Chr13: 12189110-12337063	*RNA binding motif single stranded interacting protein 3* (*rbms3*)
	spo11	Chr10: 18325018-18352338	*SPO11 initiator of meiotic double stranded breaks* (*spo11*)
Steelhead trout	sec31a	Chr5: 38498124-38527425	*SEC31 homolog A COPII coat complex component* (*sec31*)
	LOC110492082	Chr16: 40935937-40962882	*CDP-diacylglycerol--serine O-phosphatidyltransferase-like* (*pps1*)

Supplementary Table S3 Information of AS genes associated with complement and coagulation cascades pathways in livers of turbot, tongue sole and steelhead trout under different salinity environments.

Species	Gene id	Gene name	KEGG	Location	AS types

			name		
Turbot	serping1	*plasma protease C1 inhibitor*	C1INH	Chr8: 10857771-10862637	ES (4), MXE (1)
	plaub	*plasminogen activator urokinase b*	PLAU	Chr18: 20506653-20510360	ES (1)
	plg	*plasminogen*	PLG	Chr20: 18151522-18160883	ES (3), MXE (2)
Tongue sole	LOC103383919	*complement C3*	C3	Chr9: 12541498-12558354	ES (17), MXE (3)
	LOC103383958	*complement C3*	C3	Chr9: 12560678-12561571	ES (1)
	LOC103383921	*complement C3*	C3	Chr9: 12561811-12576127	ES (13), MXE (3)
	cd55	*complement decay-accelerating factor*	DAF	Chr11: 16003384-16013683	ES (7), MEX (1), A3SS (2)
	LOC103397976	*complement C5*	C5	Chr22: 13904744-13928907	ES (15), MXE (2)
Steelhead trout	LOC110524786	*complement factor H*	FH	Chr5: 84826174-84852965	ES (5), MXE (1)
	c5	*complement C5*	C5	Chr6: 44600216-44661614	ES (8)
	c8a	*complement component C8 alpha chain precursor*	C8	Chr2: 77130207-77143479	ES (1)
	LOC110530587	*complement C3*	C3	Chr8: 70002131-70061129	ES (17), MXE (5)
	LOC100653464	*complement factor B/C2*	B/C2	Chr8: 70068819-70102484	ES (9), MXE (3), A3SS (1)
	LOC100135924	*plasma protease C1 inhibitor*	C1INH	Chr10: 53808164-53840270	ES (1)
	LOC110503756	*alpha-2-macroglobulin*	A2M	Chr24: 11504145-11513502	ES (1)
	LOC110503754	*alpha-2-macroglobulin*	A2M	Chr24: 11552545-11587090	ES (7), MXE (4), A3SS (1), A5SS (1)
	LOC110507159	*alpha-2-*	A2M	Chr27:	ES (6), MXE

		macroglobulin	14542020-14574314	(1), A5SS (1)

The number of AS events in each gene were listed in the brackets of AS types.

Supplementary Table S5 Summary of 10 common DAS genes identified in livers of turbot, tongue sole and steelhead trout under different salinity environments.

Gene name	Biological function	Species	Gene ID	DAS types
heterogeneous nuclear ribonucleoprotein M (hnrnpm)	RNA splicing	Turbot	hnrnpm	ES
		Tongue sole	hnrnpm	ES
		Steelhead trout	LOC110524230	ES
cugbp Elav-like family member 1 (*celf1*)	RNA splicing	Turbot	*celf1*	A3SS
		Tongue sole	*celf1*	IR
		Steelhead	LOC110506308	A3SS
EWS RNA-binding protein 1b (*ewsr1b*)	transcriptional regulation	Turbot	LOC118313744	ES
		Tongue sole	LOC103398620	ES
		Steelhead	LOC110536095	ES
zinc finger protein 740a (*znf740a*)	transcriptional regulation	Turbot	znf740a	ES, MXE
		Tongue sole	LOC103386057	ES, MXE, A5SS
		Steelhead	LOC110528212	ES
src substrate cortactin (*cttn*)	cytoskeleton regulation	Turbot	LOC118301898	MXE
		Tongue sole	cttn	ES
		Steelhead	LOC110526157	ES
MAP/microtubule affinity-regulating kinase 2 (*mark2*)	signal transduction	Turbot	mark2	ES, A3SS,
		Tongue sole	mark2	ES
		Steelhead	LOC110509965	ES
TOM1-like protein 2 (*tom1l2*)	protein transport	Turbot	*tom1l2*	ES
		Tongue sole	LOC103382327	ES
		Steelhead	LOC110502906	IR
inter-alpha-trypsin inhibitor heavy chain H3 (*itih3*)	hyaluronan metabolism	Turbot	LOC118310142	ES
		Tongue sole	LOC103385810	ES, MXE
		Steelhead	LOC110528433	ES
reticulon 4	endoplasmic reticulum formation	Turbot	rtn4a	ES
		Tongue sole	rtn4a	ES
		Steelhead	rtn4	ES

unconventional myosin-Ib (*moy1b*)	neuronal development	Turbot	LOC118282984	ES
		Tongue sole	myo1b	ES
		Steelhead	LOC110520290	ES

Supplementary Table S6 GO enrichment analysis of DAS genes in livers of turbot, tongue sole and steelhead trout under different salinity environments.

GO term ID	Description	Species	P-value	Gene ID
GO:0030029	actin filament-based process	Turbot	0.000	pkn1a/iqsec2b/LOC118310584/actn1/grk4/LOC118287388/mical2a/LOC118301898/slka/LOC118302716/trip10a/celf1/arhgef18a/cln3/raph1b/tjp1b/LOC118310358/foxp1b/baiap2b/pacsin2/aff4/qkia/LOC118310712/scrib/elmo2/LOC118310104/dnm3a/bcar1/mlpha/abi2b/mad2l2/LOC118289364/eps8l2/tpm1/kras/gab1/llgl2/apbb2b/ankrd12/LOC118306358/LOC118310595/add1/iqgap2/cdc42bpb/vps28/LOC118300637/LOC118316914/macf1a/mtmr1a/trappc9/LOC118310583/shroom1/espn
		Tongue sole	0.000	hspg2/LOC103377718/nav2/eif4e2/pdgfra/LOC103390102/kif5b/LOC103396800/apbb2/abi1/LOC103382316/nck1/LOC103385960/cln3/nphs1/cttn/LOC103393974/daam1/myo1b/l1cam/LOC103381911/LOC103384525/sptan1/LOC103388894/actn1/sbf2/LOC103388490/LOC103390621/gab1/celf1/cflar/mlph/espnl/LOC103388292/LOC103388342/nisch/LOC103384725/myo9b/LOC103386740/LOC103391840
GO:0016325	oocyte microtubule cytoskeleton organization	Turbot	0.000	LOC118291231/mark3a/LOC118300599/LOC118310358/clasp2/ptbp1a/llgl2/LOC118310583/mark2b/rab41/LOC118316617
		Tongue sole	0.000	kif5b/clasp2/mark3/LOC103390366/ptbp2/mark2/nfe2l1
GO:1903311	regulation of mRNA metabolic	Turbot	0.000	LOC118284804/sltm/srsf2a/srpk1a/celf1/srsf7a/srsf3a/LOC118320530/rbm27/srsf1b/qkia/dyrk1ab/mbnl3/m

	process	Steelhead trout	0.000	bnl1/prpf39/srek1/ptbp1a/srrm1/LOC118309155/nup98/srsf5a/fubp3/thrap3b/hnrnpa1b/LOC118287096/fubp1/nudt21/LOC118286151/rbm17/u2af2a/LOC118317660/fxr1/rbm10 LOC110494275/sfrs3/LOC110525567/LOC110488571/LOC110523512/LOC110505280/LOC110504031/LOC110522314/LOC110499427/LOC110524090/LOC110538542/LOC110537832/LOC110532160/LOC110529920/LOC100305135/LOC110523584/LOC110538324/LOC110528326/LOC110506308/LOC110524572/LOC110520493/LOC110489035/LOC110494353/LOC110533689/LOC110527657/LOC110499704
GO:0043484	regulation of RNA splicing	Turbot	0.000	LOC118319098/LOC118291231/srsf2a/srpk1a/celf1/srsf7a/srsf3a/LOC118320530/srsf1b/qkia/dyrk1ab/mbnl3/mbnl1/hnrnph1/prpf39/srek1/ptbp1a/srrm1/LOC118309155/nup98/srsf5a/fubp3/thrap3b/hnrnpa1b/fubp1/rbm17/u2af2a/LOC118317660/rbm10
		Steelhead trout	0.000	LOC110530183/LOC110494275/sfrs3/LOC110525567/LOC110505280/LOC110504031/LOC110499427/LOC110524090/LOC110538542/LOC110537832/LOC110529920/LOC110523584/LOC110538324/LOC110528326/LOC110506308/LOC110520493/LOC110489035/LOC110494353/LOC110533689/LOC110527657
GO:0006397	mRNA processing infection	Turbot	0.000	saggf1/adar/ncoa5/LOC118291231/srsf2a/srpk1a/celf1/srsf7a/srsf3a/srsf1b/gtf2h3/mbnl1/hnrnph1/prpf39/zc3h11a/srek1/u2surp/ptbp1a/srrm1/LOC118309155/sfpq/luc7l3/srsf5a/fubp3/mthfsd/hnrnpa1b/LOC118287096/zfr/hnrnpm/nkap/sf3b1/cstf2/fubp1/nudt21/LOC118286151/rbm17/u2af2a/LOC118317660/LOC118314624/luc7l/rbm10

		Steelhead trout	0.000	fus/LOC110530183/LOC110488344/LOC110494275/LOC110524230/sfrs3/sart3/LOC110525567/LOC110488571/LOC110510479/LOC110522242/LOC110499427/LOC110524090/LOC110538542/LOC110532160/LOC110529920/LOC100305135/LOC110535465/LOC110523584/LOC110528232/LOC110528326/LOC110512933/LOC110506308/LOC110520493/LOC110534052/LOC110494353/LOC110493641/LOC110530876/LOC110527657/LOC110499704
GO:0050684	regulation of mRNA processing	Turbot	0.000	sltm/srsf2a/srpk1a/celf1/srsf7a/srsf3a/LOC118320530/rbm27/srsf1b/qkia/dyrk1ab/mbnl3/mbnl1/prpf39/srek1/ptbp1a/srrm1/LOC118309155/nup98/srsf5a/fubp3/thrap3b/hnrnpa1b/fubp1/nudt21/rbm17/u2af2a/LOC118317660/fxr1/rbm10
		Steelhead trout	0.000	LOC110494275/sfrs3/LOC110525567/LOC110505280/LOC110504031/LOC110499427/LOC110524090/LOC110538542/LOC110537832/LOC110529920/LOC110523584/LOC110538324/LOC110528326/LOC110506308/LOC110520493/LOC110489035/LOC110494353/LOC110533689/LOC110527657/LOC110499704
GO:0048024	regulation of mRNA splicing, via spliceosome	Turbot	0.000	srsf2a/srpk1a/celf1/srsf7a/srsf3a/LOC118320530/srsf1b/qkia/dyrk1ab/mbnl3/mbnl1/prpf39/srek1/ptbp1a/srrm1/LOC118309155/nup98/srsf5a/fubp3/thrap3b/hnrnpa1b/fubp1/rbm17/u2af2a/LOC118317660/rbm10
		Steelhead trout	0.000	LOC110494275/sfrs3/LOC110525567/LOC110505280/LOC110504031/LOC110499427/LOC110524090/LOC110538542/LOC110537832/LOC110529920/LOC110523584/LOC110538324/LOC110528326/LOC110506308/LOC110520493/LOC110489035/LOC110494353/LOC110533689/LOC110527657

GO:0003729	mRNA binding	Turbot	0.000	cpeb4b/sltm/LOC118291231/srsf2a/LOC118302249/celf1/srsf7a/srsf3a/rbm27/srsf1b/qkia/msi2b/hnrnph1/LOC118308924/srek1/eif4g2b/u2surp/ptbp1a/csde1/sfpq/nup98/luc7l3/srsf5a/LOC118282632/fubp3/tp53/hnrnpa1b/LOC118287096/hnrnpm/LOC118282889/ddx3xb/eif4enif1/sf3b1/rarab/cstf2/fubp1/nudt21/LOC118286151/u2af2a/LOC118317660/fxr1/luc7l/rbm10
		Steelhead trout	0.000	fus/LOC110530183/LOC110494275/LOC110524230/sfrs3/sart3/LOC110505280/LOC110504031/LOC110499427/LOC110524090/LOC110538542/LOC110537832/LOC110532160/LOC110529920/LOC100305135/LOC110498979/LOC110535465/LOC110493954/LOC110502281/LOC110523584/LOC110537820/LOC110538324/LOC110528326/LOC110528493/LOC110506308/LOC110524572/LOC110520493/LOC110494353/LOC110533689/LOC110527657/LOC110527980/LOC110499704
GO:0000381	regulation of alternative mRNA splicing, via spliceosome	Turbot	0.000	srsf2a/celf1/srsf7a/LOC118320530/srsf1b/qkia/dyrk1ab/mbnl3/mbnl1/prpf39/srek1/ptbp1a/srrm1/LOC118309155/srsf5a/fubp3/thrap3b/hnrnpa1b/fubp1/rbm17/u2af2a/LOC118317660/rbm10
		Steelhead trout	0.000	LOC110494275/LOC110525567/LOC110505280/LOC110504031/LOC110499427/LOC110524090/LOC110538542/LOC110529920/LOC110523584/LOC110538324/LOC110506308/LOC110520493/LOC110489035/LOC110527657
GO:0008380	RNA splicing	Turbot	0.000	aggf1/ncoa5/LOC118291231/srsf2a/srpk1a/celf1/srsf7a/srsf3a/srsf1b/mbnl1/hnrnph1/prpf39/srek1/u2surp/ptbp1a/srrm1/LOC118309155/sfpq/luc7l3/srsf5a/mthfsd/hnrnpa1b/LOC118287096/zfr/hnrnpm/nkap/sf3b1/

				cstf2/nudt21/LOC118286151/rbm17/u2af2a/LOC118317660/LOC118314624/luc7l/rbm10
		Tongue sole	0.000	LOC103385713/igf2bp3/cdc40/dhx38/srsf7/LOC103389920/ddx23/srsf10/LOC103386831/LOC103390366/hnrnpm/hnrnpk/ptbp2/celf1/ddx42/hnrnph1/tra2a/hnrnpr/LOC103379130/tsen2/LOC103378756
		Steelhead trout	0.000	fus/LOC110530183/LOC110488344/LOC110494275/LOC110524230/sfrs3/sart3/LOC110525567/LOC110488571/LOC110499427/LOC110524090/LOC110538542/LOC110532160/LOC110529920/LOC100305135/LOC110535465/LOC110528326/LOC110512933/LOC110506308/LOC110520493/LOC110534052/LOC110494353/LOC110493641/LOC110530876/LOC110527657
GO:0000375	RNA splicing, via transesterification reactions	Turbot	0.000	aggf1/ncoa5/LOC118291231/srsf2a/srpk1a/celf1/srsf7a/srsf3a/srsf1b/mbnl1/hnrnph1/prpf39/srek1/u2surp/ptbp1a/srrm1/LOC118309155/sfpq/luc7l3/srsf5a/mthfsd/hnrnpa1b/LOC118287096/zfr/hnrnpm/nkap/sf3b1/cstf2/nudt21/LOC118286151/rbm17/u2af2a/LOC118317660/LOC118314624/luc7l/rbm10
		Tongue sole	0.000	LOC103385713/igf2bp3/cdc40/dhx38/srsf7/LOC103389920/ddx23/srsf10/LOC103386831/LOC103390366/hnrnpm/hnrnpk/ptbp2/celf1/ddx42/hnrnph1/tra2a/hnrnpr/LOC103379130/LOC103378756
		Steelhead trout	0.000	fus/LOC110530183/LOC110488344/LOC110494275/LOC110524230/sfrs3/sart3/LOC110525567/LOC110488571/LOC110499427/LOC110524090/LOC110538542/LOC110532160/LOC110529920/LOC100305135/LOC110535465/LOC110528326/LOC110512933/LOC110506308/LOC110520493/LOC110534052/LOC110494353/LO

				C110493641/LOC110530876/LOC110527657
		Turbot	0.000	aggf1/ncoa5/LOC118291231/srsf2a/srpk1a/celf1/srsf7a/srsf3a/srsf1b/mbnl1/hnrnph1/prpf39/srek1/u2surp/ptbp1a/srrm1/LOC118309155/sfpq/luc7l3/srsf5a/mthfsd/hnrnpa1b/LOC118287096/zfr/hnrnpm/nkap/sf3b1/cstf2/nudt21/LOC118286151/rbm17/u2af2a/LOC118317660/LOC118314624/luc7l/rbm10
GO:0000377	RNA splicing, via transesterification reactions with bulged adenosine as nucleophile	Tongue sole	0.000	LOC103385713/igf2bp3/cdc40/dhx38/srsf7/LOC103389920/ddx23/srsf10/LOC103386831/LOC103390366/hnrnpm/hnrnpk/ptbp2/celf1/ddx42/hnrnph1/tra2a/hnrnpr/LOC103379130/LOC103378756
		Steelhead trout	0.000	fus/LOC110530183/LOC110488344/LOC110494275/LOC110524230/sfrs3/sart3/LOC110525567/LOC110488571/LOC110499427/LOC110524090/LOC110538542/LOC110532160/LOC110529920/LOC100305135/LOC110535465/LOC110528326/LOC110512933/LOC110506308/LOC110520493/LOC110534052/LOC110494353/LOC110493641/LOC110530876/LOC110527657
		Turbot	0.000	aggf1/ncoa5/LOC118291231/srsf2a/srpk1a/celf1/srsf7a/srsf3a/srsf1b/mbnl1/hnrnph1/prpf39/srek1/u2surp/ptbp1a/srrm1/LOC118309155/sfpq/luc7l3/srsf5a/mthfsd/hnrnpa1b/LOC118287096/zfr/hnrnpm/nkap/sf3b1/cstf2/nudt21/LOC118286151/rbm17/u2af2a/LOC118317660/LOC118314624/luc7l/rbm10
GO:0000398	mRNA splicing, via spliceosome	Tongue sole	0.000	LOC103385713/igf2bp3/cdc40/dhx38/srsf7/LOC103389920/ddx23/srsf10/LOC103386831/LOC103390366/hnrnpm/hnrnpk/ptbp2/celf1/ddx42/hnrnph1/tra2a/hnrnpr/LOC103379130/LOC103378756
		Steelhea	0.000	fus/LOC110530183/LOC110488344/

		d trout		LOC110494275/LOC110524230/sfrs3/sart3/LOC110525567/LOC110488571/LOC110499427/LOC110524090/LOC110538542/LOC110532160/LOC110529920/LOC100305135/LOC110535465/LOC110528326/LOC110512933/LOC110506308/LOC110520493/LOC110534052/LOC110494353/LOC110493641/LOC110530876/LOC110527657

Supplementary Table S7 KEGG enrichment analysis of DAS genes in livers of turbot, tongue sole and steelhead trout under different salinity environments.

Pathway ID	Pathway description	Species	P-value	Gene ID
KO04610	Complement and coagulation cascades	Turbot	0.004	spint2/im:7151449/serpinf2a/f7l/LOC118318093/serpinc1/cfi/LOC118310519/LOC118310521/LOC118297752/LOC118287884
		Steelhead trout	0.012	cfi/LOC110533697/LOC110507159/masp1/LOC110504649/LOC110525399/LOC110503754/LOC110524099
KO00564	Glycerophospholipid metabolism	Turbot	0.019	LOC118289108/plpp1a/pnpla7b/pemt/chka/si:ch73-21k16.5/dgkza/etnk1/gpcpd1/lpcat3
		Steelhead trout	0.002	LOC110503015/LOC110494040/chkb/LOC110536310/LOC110523465/LOC110520584/LOC110534067/LOC110504186/LOC110523914
KO05168	Herpes simplex virus 1 infection	Turbot	0.010	srsf2a/ep300a/srpk1a/srsf7a/srsf3a/LOC118283298/srsf1b/fkbp5/rela/socs2/srsf5a/tp53/LOC118318615/LOC118302761/ptpn11b/jak2a/tp63
		Tongue sole	0.047	LOC103384730/LOC103394065/LOC103393270/LOC103383760/srsf7/LOC103396400/hnrnpk/nfkb2/LOC103382011/LOC103384375
		Steelhead trout	0.005	LOC100135915/LOC110493986/LOC110502378/LOC110509930/sfrs3/LOC110501897/LOC110499427/LOC110501624/LOC110538542/LOC110537832/LOC100301644/LOC110528326/LOC110532763/LOC110494353

				/LOC100135905
KO03040	Spliceosome	Turbot	0.004	srsf2a/srsf7a/srsf3a/srsf1b/u2surp/LOC118309155/srsf5a/hnrnpa1b/LOC118287096/hnrnpm/sf3b1/rbm17/u2af2a
		Tongue sole	0.002	cdc40/dhx38/srsf7/LOC103378016/ddx23/srsf10/hnrnpm/hnrnpk/ddx42/tra2a
		steelhead trout	0.000	LOC110494224/LOC110524230/sfrs3/LOC110525567/LOC110499427/LOC110538542/LOC110529920/LOC110535465/LOC110528326/LOC110512933/LOC110520493/LOC110534052/LOC110494353/ppip5k2/LOC110530711
KO05216	Thyroid cancer	Turbot	0.002	LOC118291231/rxraa/ptbp1a/tpm1/kras/tp53/rxrbb/rxrba/tp63
		Tongue sole	0.034	tpr/LOC103390366/ptbp2/tcf7l2/LOC103397252
KO05202	Transcriptional misregulation in cancer	Turbot	0.010	zbtb44/LOC118291231/rxraa/eya3/etv5a/ncor1/ccnd3/rela/ptbp1a/tcf3a/ewsr1b/tp53/bcl6ab/ldb1b/rxrbb/rarab/rxrba/elf2a/prom1b/tp63/spi1b
		steelhead trout	0.008	LOC110520691/fus/LOC110502378/LOC110530183/LOC110500077/LOC110504828/LOC110509930/LOC110536095/erg/LOC110537494/LOC110504926/LOC110524090/LOC100136604/LOC110525034/atm/LOC100135905

Supplementary Table S8 Detailed information of 12 common DAS genes associated with RNA processing in livers of turbot, tongue sole and steelhead trout under different salinity environments.

Gene name	Species	Gene ID	Location
heterogeneous nuclear ribonucleoprotein M (*hnrnpm*)	Turbot	LOC118317783	NC_049699.1: 12473543-12481354
	Tongue sole	LOC103393912	NC_024308.1: 2792611-2799398
	Steelhead trout	LOC110524230	NC_035081.1: 57385663-57400932
cugbp Elav-like family member 1	Turbot	LOC118302421	NC_049692.1: 26408334-26440676
	Tongue sole	LOC103379022	NC_024311.1: 10300854-10317431

(*celf1*)	Steelhead trout	LOC110506308	NC_035102.1: 7328525-7371029
ELAV-like protein 1 (*elavl1*)	Turbot	LOC118286151	NC_049704.1: 5769398-5777501
	Tongue sole	LOC103379130	NC_024307.1: 11309034-11315441
serine and arginine rich splicing factor 7a (*srsf7a*)	Turbot	LOC118295981	NC_049690.1: 5304167-5312513
	Tongue sole	LOC103386057	NC_024317.1: 5095505-5114743
heterogeneous nuclear ribonucleoprotein A1b (*hnrnpa1b*)	Turbot	LOC118317579	NC_049698.1: 14269432-14273666
	Steelhead trout	LOC110488571	NC_035090.1: 46064008-46066926
far upstream element-binding protein 3 (*fubp3*)	Turbot	LOC118285005	NC_049703.1: 9908244-9928182
	Steelhead trout	LOC110523584	NC_035081.1: 26846059-26872297
U2 snRNP-associated SURP motif-containing protein (*u2surp*)	Turbot	LOC118290873	NC_049708.1: 1322730-1331172
	Steelhead trout	LOC110535465	NC_035087.1: 18441219-18458858
serine and arginine rich splicing factor 3a (*srsf3a*)	Turbot	LOC118309006	NC_049693.1: 12184237-12195705
	Steelhead trout	LOC110528326	NC_035083.1: 58680534-58680661
serine and arginine rich splicing factor 2a (*srsf2a*)	Turbot	LOC118287600	NC_049705.1: 17262905-17266021
	Steelhead trout	LOC110499427	NC_035096.1: 33094603-33099399
	Steelhead trout	LOC110538542	NC_035088.1: 76243389-76246519
polypyrimidine tract-binding protein 1 (*ptbp1*)	Turbot	LOC118285727	NC_049704.1: 6420166-6434631
	Steelhead trout	LOC110524090	NC_035081.1: 52966746-52992075
RNA-binding protein 39 (*rbm39*)	Tongue sole	LOC103386831	NC_024317.1: 20223728-20229142
	Steelhead trout	LOC110527657	NC_035083.1: 26125980-26184997
polypyrimidine tract-binding protein 2 (*ptbp2*)	Tongue sole	LOC103376868	NC_024309.1: 732989-748884
	Steelhead trout	LOC110530183	NC_035084.1: 51612791-51631862

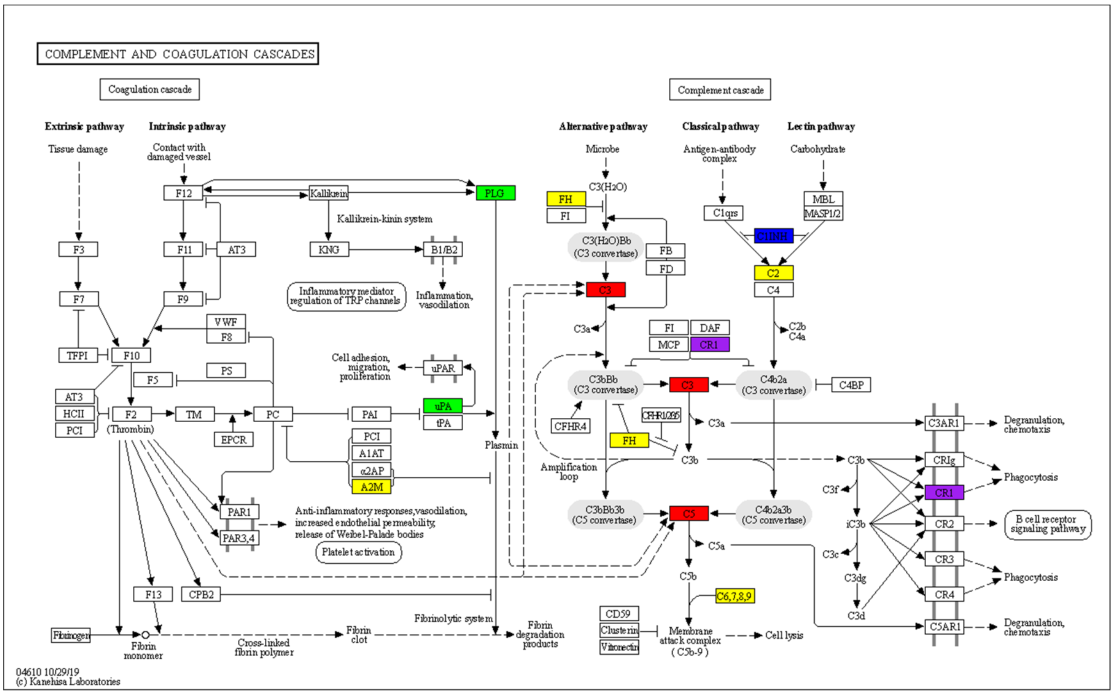

Supplementary Figure S1 Complement and coagulation cascades pathway of AS genes located in the hotspot regions in livers of turbot, tongue sole and steelhead trout. AS genes in turbot, tongue sole and steelhead trout were showed with green, purple and yellow boxes, respectively. The common AS genes in both steelhead trout and tongue sole were marked with red boxes. Blue box represented the common AS genes in turbot and steelhead trout.

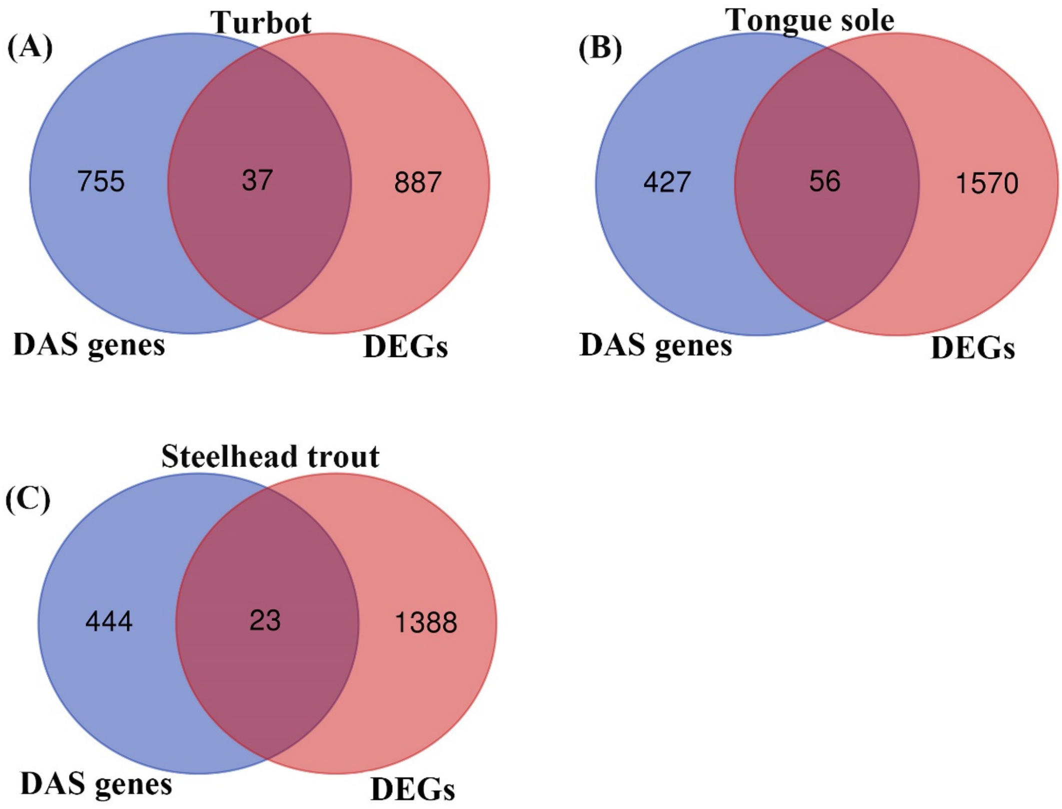

Supplementary Figure S2 Veen diagrams showing the numbers of common DEGs and DAS genes in livers of turbot (A), tongue sole (B) and steelhead trout (C) under different salinity environments.

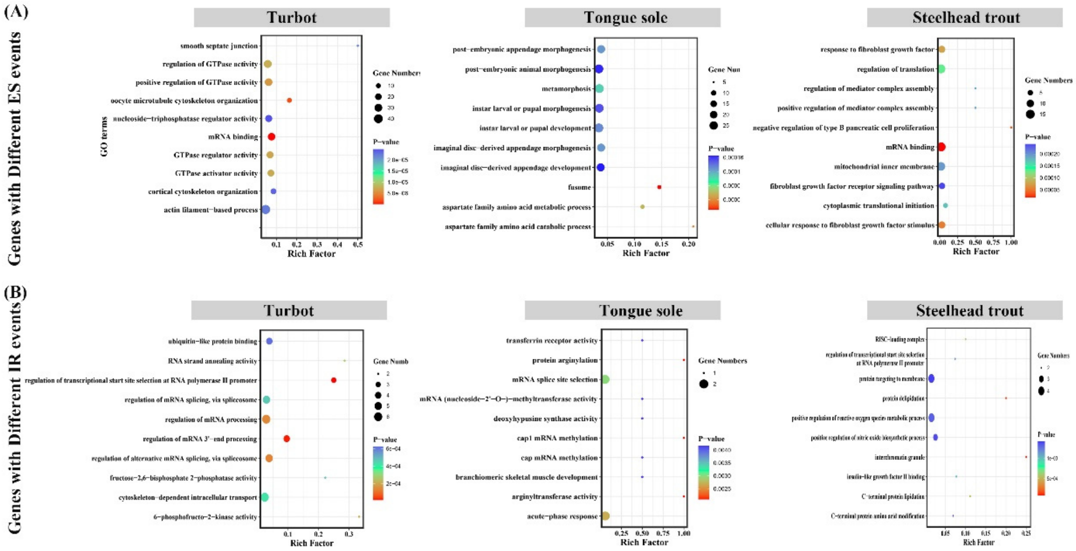

Supplementary Figure S3 GO enrichment analysis of different ES (A) and IR (B) genes in livers of turbot, tongue sole and steelhead trout under different salinity environments.

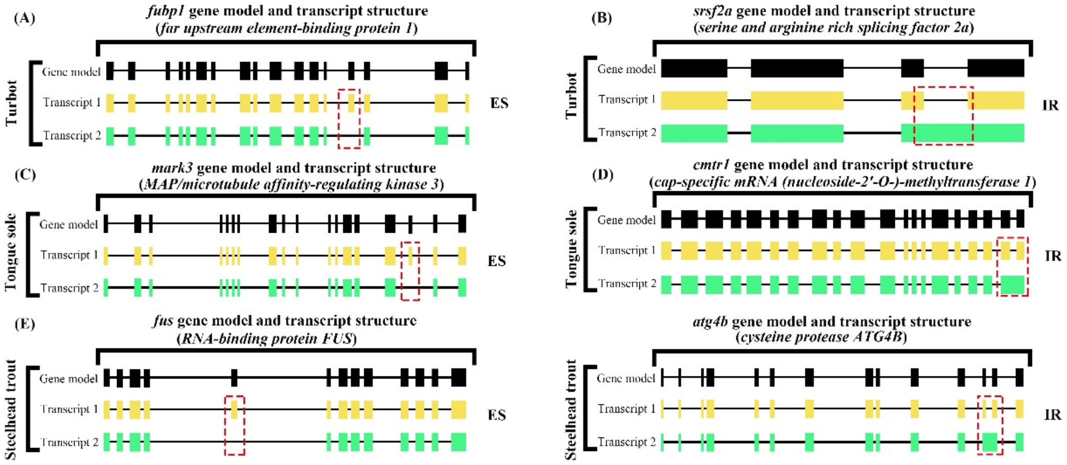

Supplementary Figure S4 Splicing models of differential ES and IR events in livers of turbot (A-B), tongue sole (C-D) and steelhead trout (E-F) under different salinity environments. Filled boxed represented exons and introns were showed using black lines.

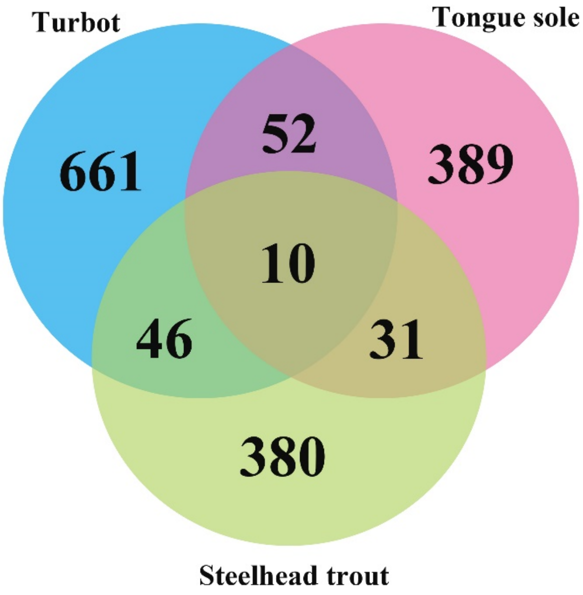

Supplementary Figure S5 Venn diagrams showing the numbers of common DAS genes in livers of turbot, tongue sole and steelhead trout under different salinity environments.

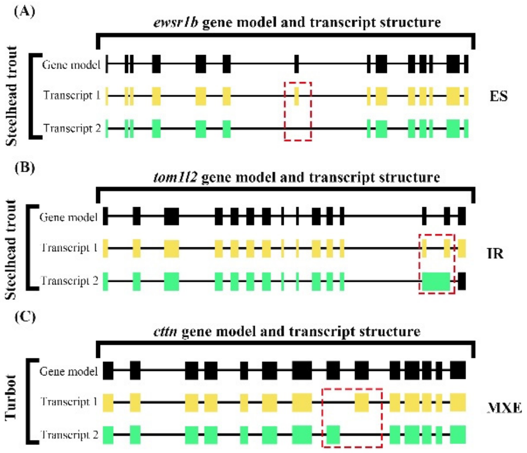

Supplementary Figure S6 Splicing models of common DAS genes in livers of turbot, tongue sole and steelhead trout under different salinity environments. Filled boxed represented exons and introns were showed using black lines.

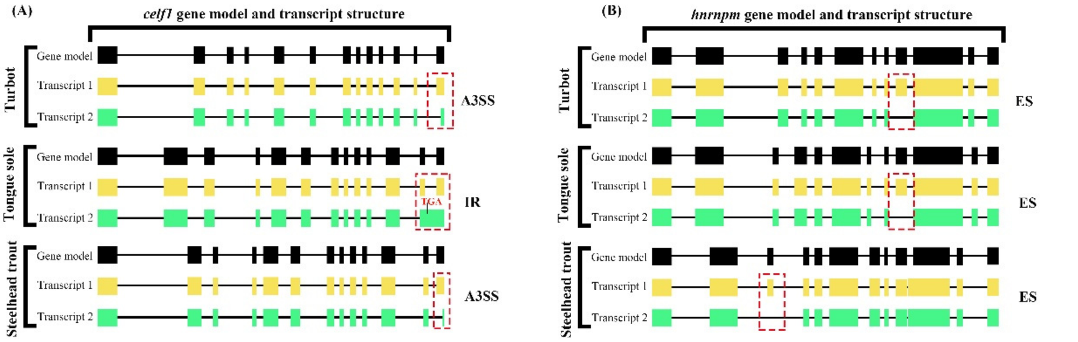

Supplementary Figure S7 Splicing models of common DAS genes associate with RNA splicing in livers of turbot, tongue sole and steelhead trout under different salinity environments. Filled boxed represented exons and introns were showed using black lines.
